# Olfactory Ensheathing Cell Tumor Arising from the Olfactory Mucosa

**DOI:** 10.1155/2012/426853

**Published:** 2012-05-24

**Authors:** Eriko Ogino-Nishimura, Takayuki Nakagawa, Yoshiki Mikami, Juichi Ito

**Affiliations:** ^1^Department of Otolaryngology, Head and Neck Surgery, Graduate School of Medicine, Kyoto University, Kyoto 606-8507, Japan; ^2^Department of Diagnostic Pathology, Kyoto University Hospital, Kyoto 6060-8507, Japan

## Abstract

We report a rare case of olfactory ensheathing cell tumor. A female presented a large soft mass extending medially to the olfactory cleft and laterally to the middle meatus in the left nasal cavity. Imaging studies confirmed a cystic mass extending superiorly into the frontal lobe, indicating that the tumor arouse from the olfactory mucosa. A subtotal resection was achieved through an endoscopic endonasal approach without operative complications. Immunohistochemically constituent cells were diffusely positive for S-100 protein, but olfactory ensheathing cell tumor was diagnosed by negative staining for Leu7 (CD57). This case indicates that olfactory ensheathing cell tumor should be included in differential diagnoses for the olfactory cleft tumors.

## 1. Introduction

Rare examples of olfactory schwannomas have been previously reported [1–24], but their histogenesis appears enigmatic because olfactory nerves lack a Schwann cell layer. Recently, the concept of olfactory ensheathing cell tumor was proposed for those tumors previously considered to be schwannomas located at the skull base [25–29]. Absence of immunostaining for Leu7 (CD57) is considered to distinguish olfactory ensheathing cell tumors from schwannomas, which are typically positive for this particular marker. However, there are few case reports of subfrontal schwannomas that note Leu7 staining has been undertaken. Therefore, further accumulation of case reports is needed to establish definitive diagnosis of olfactory ensheathing cell tumors. Herein we report the first case of olfactory ensheathing cell tumor arising from the olfactory cleft with intracranial extension.

## 2. Case Presentation

A 41-year-old-female had complained of headache and loss of olfactory function and underwent consultation at the Department of Otolaryngology of a general hospital. Endoscopic examination revealed a large mass involving the olfactory cleft of the left nasal cavity. Microscopic examination of a biopsy specimen indicated a diagnosis of neurogenic tumor. She was then referred to Kyoto University Hospital for further examination and treatment.

**Figure 1:**
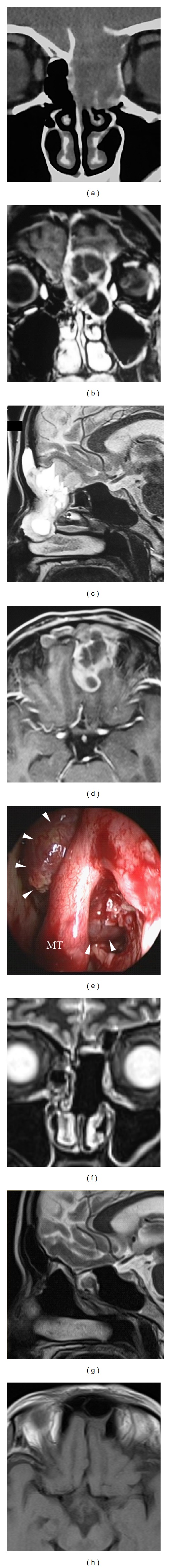
Preoperative computed tomography (a), and magnetic resonance images ((b) coronal, (c) sagittal, and (d) axial) show a cystic mass extending from the left olfactory cleft to the olfactory groove. An endoscopic image (e) shows the tumor extending from the olfactory cleft to the middle meatus (arrowheads). MT: middle turbinate. Magnetic resonance images two years after operation show no recurrence of tumors (f)–(h).

Nasal endoscopy demonstrated a soft, whitish mass occupying the olfactory cleft and extending laterally to the middle meatus, with destruction of the middle turbinate in the left nostril. Computed tomography (CT) displayed a lesion at the olfactory cleft that extended superiorly to the olfactory groove, with a bone defect in the skull base (Figure 1(a)). The cribriform plate was elevated upward indicating that the tumor originated from the extracranial compartment. Magnetic resonance imaging (MRI) revealed a mass showing cystic changes (Figures 1(b)–1(d)), with solid portions demonstrating strong postgadolinium contrast enhancement. Our initial diagnosis based on radiographic findings was esthesioneuroblastoma. Partial resection of the tumor in the olfactory cleft was then performed using the endoscopic endonasal approach under local anesthesia, which suggested a histopathologic diagnosis of schwannoma. Based on this result, we planned subtotal resection of the tumor via an endoscopic endonasal approach.

Under general anesthesia, the uncinate process in the left nostril was removed to expose the tumor in the middle meatus (Figure 1(e)). The tumor was attached but had not invaded the internal orbital wall. The anterior part of the middle turbinate was separated from the agger nasi and reserved into the choana as a pedicle flap. Resection of the agger nasi using a drill resulted in exposure of the entire anterior surface of the tumor. A tumor capsule was dissected from the nasal septum and then from the crista galli using a suction elevator and a 45-degree angled endoscope for visualization. During this procedure, the olfactory nerves were identified. After confirmation of their location, the anterior and lateral walls of the tumor were dissected from the posterior wall of the frontal sinus and nasofrontal duct.

Afterward, the tumor capsule at the anterior surface was opened using an ultrasonic cutter (Harmonic scalpel, EthiconEndo-Surgery, Blue Ash, OH). The tumor contents were debulked with an ultrasonic surgical aspiration (CUSA, Tyco Healthcare Radionics, Burlington, MA) without bleeding. The tumor capsule was resected, except for the region connected to the dura matter. Following surgery, the region with the bone defect was covered with a mucoperiosteal pedicle flap that originated from the nasal septum and the middle turbinate. Pedicle flaps were fixed with fibrin glue, then covered with pieces of gelatin sponges. The operation lasted 3 h, and the volume of blood loss was less than 10 mL.

**Figure 2:**
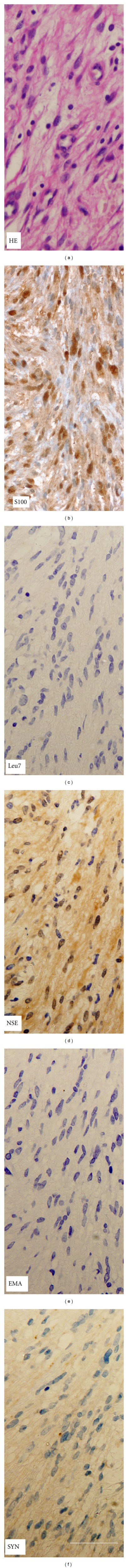
Hematoxylin and eosin (HE) staining shows that the tumor is composed of spindle cells with eosinophilic cytoplasm (a). Immunohistochemistry demonstrates that the tumor is strongly positive for S100 (b) and neuron-specific enolase (NSE, (d)), weakly positive for synaptophysin (SYN, (f)), and is negative for Leu7 (c) and epithelial membrane antigen (EMA, (e)). In immunohistochemistry, nuclear staining with hematoxylin was performed. Scale bar in (f) represents 50 *μ*m for (a)–(f).

Microscopic examination of the resected tumor demonstrated a neoplasm composed of spindle cells with eosinophilic cytoplasms and elongated or wavy nuclei with occasional symplastic changes (Figure 2(a)). The mitotic index was less than one per ten high-power fields, and there was no geographic tumor necrosis. All features were apparently compatible with schwannomas. Immunohistochemically, the spindle cells were diffusely positive for S-100 protein, neuron-specific enolase, and synaptophysin (Figures 2(b), 2(d), and 2(f)), and negative for epithelial membrane antigen (Figure 2(e)), which also supported the diagnosis of schwannoma, although Leu7 was completely negative (Figure 2(c)). The Ki-67 labeling index was 2%. These results were considered to best fit with a diagnosis of olfactory ensheathing cell tumor.

No perioperative cerebrospinal leakage was identified. Postoperative imaging examinations confirmed subtotal extirpation of the tumor. The patient had an uneventful postoperative course, and no further recurrence was detected during the 2-year follow-up period (Figures 1(f)–1(h)).

## 3. Discussion

Twenty-four cases of schwannoma located at the olfactory nerve have previously been reported as olfactory schwannomas, olfactory groove schwannomas, or subfrontal schwannomas. The pathogenesis of these tumors has been a matter of controversy, and there are several hypotheses concerning their putative origin. Developmental theories indicate a derivation from aberrant Schwann cells in the central nervous system. Alternatively, anterior ethmoidal nerves or the meningeal branches of the trigeminal nerve, in which Schwann cells reside, have been suggested as an origin in some reports [5, 13, 14, 18]. Another suggested origin is the olfactory ensheathing cells, glial cells that ensheath the axons of the first cranial nerve [30]. These cells have properties of Schwann cells in that they promote and assist in the growth of axons.

The first study about olfactory ensheathing cell tumor was published by Yasuda et al. in 2006, and five cases have been reported thereafter to date [25–29]. Olfactory ensheathing cell tumor and schwannomas share morphological features as well as S-100 protein immunopositivity, although Leu7 is usually negative in olfactory ensheathing cell tumor and positive in cases of schwannoma [31]. In this context, the tumor in the present study best fits a diagnosis of olfactory ensheathing cell tumor. However, information on Leu7 reactivity in cases of subfrontal schwannoma is limited [5, 21, 22], and 20% of tumors considered to be schwannomas are, in fact, Leu7 negative [32]. Therefore, further studies are necessary to fully characterize olfactory ensheathing cell tumor and its normal counterpart.

All olfactory ensheathing cell tumors in previous case reports [25–29] were located in intracranial regions indicating that they originated from the olfactory bulb because olfactory ensheathing cells lie in the olfactory mucosa and olfactory bulb, and surround axons of olfactory mucosa and olfactory bulb [33]. In the present case, radiographic findings indicated the tumor originated from the olfactory mucosa. To our knowledge, this is the first case of olfactory ensheathing cell tumor arising from the olfactory mucosa. In a series of previous cases, subfrontal meningioma was the primary differential diagnosis because of the location of tumors. In the present case, radiographic findings suggested esthesioneuroblastoma as a diagnosis, and preoperative histological examination was required for differential diagnosis. Our case report suggests that olfactory ensheathing cell tumor should be included in differential diagnoses for olfactory cleft tumors.
